# The associations of leg lean mass with foot pain, posture and function in the Framingham foot study

**DOI:** 10.1186/s13047-014-0046-5

**Published:** 2014-11-12

**Authors:** Robert R McLean, Alyssa B Dufour, Patricia P Katz, Howard J Hillstrom, Thomas J Hagedorn, Marian T Hannan

**Affiliations:** Hebrew Senior Life Institute for Aging Research, Boston, MA USA; Harvard Medical School, Boston, MA USA; University of California, San Francisco, San Francisco, CA USA; Hospital for Special Surgery, New York, NY USA

**Keywords:** Lean mass, Foot pain, Foot structure, Foot function, Population-based cohort

## Abstract

**Background:**

Foot disorders are common in older adults and associated with impaired lower extremity function. Reduced muscle mass may play a role in the etiology of foot disorders and consequent poor function.

**Methods:**

We examined the association of leg lean mass with foot pain, posture and function among 1,795 individuals (mean age 67 years) from the population-based Framingham Foot Study (2002–2008). Pain was assessed via questionnaire, and a pressure mat classified foot posture (arch: high, low, referent) during standing and function (pronation, supination, referent) during gait. Leg lean mass was measured by whole body dual energy x-ray absorptiometry.

**Results:**

In age- and body mass index-adjusted logistic (pain) and multinomial logistic (posture, function) regression models, a 1-standard deviation increase in leg lean mass was associated with lower odds of foot pain (OR = 0.76, 95% CI: 0.68, 0.86) and pronation (OR = 0.76, 95% CI: 0.67, 0.85), and higher odds of supination (OR = 1.17, 95% CI: 1.04, 1.31). Adjustment for sex attenuated these associations. Higher leg lean mass was associated with lower odds of high arch, even after adjustment for sex (OR = 0.73, 95% CI: 0.60, 0.89).

**Conclusions:**

Though not related to foot pain or function, reduced leg lean mass was associated with extreme foot posture in older adults. Loss of muscle mass with aging may thus play a role in the etiology of functional impairment due to foot disorders.

## Background

Foot pain is commonly reported in the general population, affecting approximately 1 out of every 5 older adults [1]. There is growing evidence that foot pain is associated with impaired lower extremity function [2,3], balance and gait disorders [4,5], mobility disability [6,7], and falls [8–10]. Despite these observed associations, the mechanistic pathways linking foot pain with poor function and disability have not been fully elucidated. Identifying components of this pathway is important for developing targeted interventions to reduce disability resulting from foot pain.

Following the fourth decade of life, muscle mass decreases with aging, resulting in loss of muscle strength and function [11], which are associated with lower extremity functional impairment and disability in older adults [12,13]. Studies of clinical populations suggest that poor lower extremity muscle function is associated with atypical foot posture and function. Patients with posterior tibial tendon dysfunction, a common acquired flatfoot deformity, have lower ankle and hip muscle strength compared to non-patients [14], and the degree of deformity is inversely related to leg muscle strength [15]. Imbalance of the muscles that control the foot, but originate in the lower leg has been implicated in the etiology of both idiopathic and disease-related pes cavus (high arches) [16–18]. Further, evidence from studies of runners suggests that weakness in lower leg [19] and hip-stabilizing muscles [20] can lead to disturbed lower extremity mechanics, resulting in over-pronation of the foot. Such atypical foot posture and foot function have been associated with foot pain in population-based studies [21–23].

It would be of clinical interest to identify age-related loss of muscle mass as a mechanism for foot pain and consequent disability in the general population since muscle mass and function can be improved with exercise [24], and may thus be targeted for treating or preventing foot pain. Yet data linking muscle mass with foot disorders in community-based studies are limited. We are aware of only one prior study that found no association between total body skeletal muscle mass and foot pain [25], and none that have examined foot posture or function. Thus, the relation of muscle mass with foot pain, posture and function in the general population is unknown.

The purpose of this investigation was to evaluate the relation of leg lean mass with foot pain, posture and function in a population-based study of older men and women. We hypothesized that lower leg lean mass would be associated with greater risk for foot pain, planus (flat) and cavus (high) foot posture, and supinated and pronated foot function.

## Methods

### Participants

Participants in this study included members of the Framingham Foot Study, an ancillary study derived from the Framingham Study Original and Offspring cohorts. The Framingham Study was begun in 1948 with the objective of investigating risk factors for heart disease. The Original cohort enrolled 5,209 men and women, aged 28 to 62 years, who were recruited from a two-thirds sample of all residences of the town of Framingham, Massachusetts and have since been examined every two years [26]. The Offspring cohort was initiated in 1971 to determine familial risk factors for cardiovascular disease, enrolling 5,124 male and female adult children, and their spouses, of the Original cohort [27]. The age of Offspring participants ranged from 5 to 70 years at enrollment, and they have been examined approximately every four years. Between 2002 and 2008, 2,447 cohort members (266 Original, 2,181 Offspring) participated in the Framingham Foot Study, during which they were queried regarding foot pain and completed a plantar pressure and loading assessment during standing and walking. The current analysis includes the 1,795 participants (776 men, 1,019 women) who had information on leg lean mass that was obtained as part of the Framingham Osteoporosis Study in 1992–93 (Original) and in 1996–2001 (Offspring). The institutional review boards at Hebrew SeniorLife and Boston University approved the study, and all participants signed an informed consent form prior to study enrolment.

### Foot pain

Foot pain was assessed using the following National Health and Nutrition Examination Survey-based query about foot pain: “On most days, do you have pain, aching, or stiffness in either of your feet?” Possible responses were no; yes, left foot only; yes, right foot only; yes, both feet; yes, not sure what side; and unknown. Responses were collapsed into 2 groups: yes, pain in one or both feet; or no, no pain in either foot.

### Foot posture and function

A Tekscan Matscan (Tekscan Inc., Boston, Massachusetts) pressure mat was used to collect plantar pressure data at 40 Hz during walking and during a single frame snapshot of bipedal quiet stance. Walking data were collected using the two-step method, a method in which participants strike the mat with their second foot, that has been shown to be as reliable as mid-gait techniques [28]. One walking scan was collected on each foot, in addition to a scan of both feet in quiet, bipedal, weight bearing stance.

#### Foot posture

Foot posture was defined using the Modified Arch Index (MAI) calculated on each foot using the quiet stance scans. To determine the MAI value, the foot, excluding the toes, is divided into three equal parts, and the total force in the middle third is divided by the total force in all three foot regions, as previously described [23]. MAI is correlated with other measures of foot posture, notably navicular height [29]. As each participant had two MAI values (right and left), the value farthest from the median was chosen to be used in the analysis, similar to prior studies of bilateral conditions [30,31]. Participants’ foot posture was classified based on quintile cut-points from the distribution of MAI values from all Framingham Foot Study participants (3,100 participants yielding 6,153 feet; age range 36–100 years), including those not included in the current analysis [32,33]. MAI > 0.163 (highest quintile) was classified planus (low arch), MAI from 0.031 to 0.163 (middle three quintiles) was classified rectus (referent), and MAI < 0.031 was classified cavus (high arch). Rectus was considered the referent group.

#### Foot function

Foot function was characterized using participant’s walking plantar pressures with the center of pressure excursion index (CPEI), a footprint-based measure of foot function [34]. To determine the CPEI value, a line is drawn from the first and last points of each foot’s center of pressure trajectory, and the distance of the center of pressure curve at the distal third of the foot from the constructed line is recorded, as previously described [35]. This value is normalized by foot width and multiplied by 100 to obtain a percentage excursion of the center of pressure. CPEI has been shown to be sensitive to changes in clinical measures of static foot alignment [34]. Similar to the method used for MAI, CPEI was categorized into three groups: CPEI > 20.9 (highest quintile) was classified as supination, CPEI from 7.4 to 20.9 (middle three quintiles) was classified as the referent group and CPEI < 7.4 was classified as pronation.

### Dual-energy x-ray absorptiometry

Body composition measures were obtained from whole body dual-energy x-ray absorptiometry (DXA) scans ascertained using a Lunar DPX-L (LunarCorp, Madison, Wisconsin), as previously described [36]. The “legs” region of interest includes the entire lower extremities below the pelvis. Because muscle mass is strongly correlated with body height, relative leg lean mass was calculated as the lean mass of the legs region in kilograms from the DXA scan, divided by the square of body height recorded at the time of the DXA scan (kg/m^2^) [37].

### Other variables

Information on age (years), height and weight was recorded at the time of the Framingham Foot Study exam. Weight was measured using a standardized balance beam scale and recorded to the nearest half pound. Height without shoes was measured to the nearest ¼ inch using a calibrated stadiometer. Body mass index (BMI) was calculated as a participant’s weight in kilograms divided by their height in meters squared (kg/m^2^).

### Statistical analysis

Means and standard deviations of continuous variables, and frequencies of categorical variables, were calculated for all participant characteristics. T-tests and chi-square tests were used to compare continuous and categorical variables, respectively, between men and women. To determine the association between leg lean mass and foot pain (yes/no), logistic regression calculated the odds ratio (OR) and 95% confidence interval (CI) for the likelihood of reporting foot pain associated with a one standard deviation increase in leg lean mass. For the association of leg lean mass with foot posture, multinomial logistic regression analysis calculated the OR and 95% CI for planus and cavus MAI, relative to the referent, for a one standard deviation increase in leg lean mass. A similar multinomial analysis was used to determine the association of leg lean mass with foot function (OR for pronatory and supinatory versus the referent). Regression models were first unadjusted for covariates, followed by adjustment for age, and for BMI to account for adiposity, which is associated both with lean mass [38] and with foot pain [39]. Models were further adjusted for sex, and sex-specific models were examined to evaluate whether sex modified the association between leg lean mass and foot pain, posture and function. Because the associations between leg lean mass and foot pain, posture and function may not be linear, quartiles of leg lean mass were created and regression analyses were repeated to determine the likelihood of foot pain, posture and function in the lowest quartile of leg lean mass relative to the upper three quartiles combined. Results from analyses of quartiles of leg lean mass were similar, thus only results of analyses with leg lean mass as a continuous variable are reported.

The time between lean mass measurement and the subsequent foot assessment ranged from 1.5 to 11.0 years among study participants, with an average of 6.7 years. Thus it is possible that interim loss of muscle mass due to aging could impact our estimates of the associations of lean mass with foot pain, posture and function. To explore whether age-associated changes in lean mass may have influenced our results, we repeated our analyses using lean mass values adjusted to estimate each participants lean mass at the time of the foot assessment, based on the time between their lean mass and foot assessments and reports in the literature that muscle mass declines at a rate of approximately 1% per year after age 50 [40,41].

Statistical analyses were conducted using the SAS statistical analysis package, version 9.3 (SAS Institute, Cary, NC). Level of statistical significance for *P* values (two-sided) was 0.05.

## Results

Participant characteristics are listed in Table 1. Mean age of men and women was 68 years and 67 years, respectively. Compared to women, men had higher BMI (28.6 kg/m^2^ vs. 27.6 kg/m^2^, *P* < 0.0001) and greater height-adjusted leg lean mass [5.75 kg/m^2^ (SD = 0.59) vs. 4.52 kg/m^2^ (SD = 0.47), *P* < 0.0001]. A higher proportion of women (25.1%) than men (16.6%) reported foot pain (*P* < 0.0001). The difference between men and women in the proportions of cavus (men 31.6%, women 29.0%) and planus (men 29.0%, women 24.3%) foot posture did not reach statistical significance, yet the difference in proportions of foot function did (*P* < 0.0001). Women had higher prevalence of pronation (men 21.5%, women 38.4%) and lower prevalence of supination (men 38.3%, women 23.2%) compared to men.

**Table 1:** **Characteristics** ^**a**^ **of Framingham foot study participants, 2002–2008**

	**All participants**	**Men**	**Women**	***P*** **value** ^**b**^
N	1795	776	1,019	
Age (years)	67.4 (10.2)	67.9 (10.1)	67.0 (10.2)	0.08
Body mass index (kg/m^2^)	28.1 (4.9)	28.6 (4.2)	27.6 (5.4)	<0.0001
Leg lean mass (kg/m^2^)	5.05 (0.81)	5.75 (0.59)	4.52 (0.47)	<0.0001
Foot pain (%)	21.5	16.6	25.1	<0.0001
Foot posture (%)				
Cavus	30.5	29.0	31.6	0.07
Rectus	43.2	42.0	44.1	
Planus	26.4	29.0	24.3	
Foot function (%)				
Pronation	31.1	21.5	38.4	<0.0001
Referent	39.2	40.2	38.5	
Supination	29.7	38.3	23.2	

^a^Mean (SD) unless otherwise noted.

^b^ *P* values for comparison between men and women.

### Foot pain

Among all participants, a one standard deviation increase in leg lean mass was associated with 15% lower odds of foot pain in the unadjusted model (OR = 0.85, 95% CI 0.76, 0.96), and 24% lower odds after adjustment for age and BMI (OR = 0.76, 95% CI 0.67, 0.86) (Table 2). This association was, however, attenuated after additional adjustment for sex (OR = 0.89, 95% CI: 0.73, 1.08). Leg lean mass was not associated with foot pain in sex-specific analyses.

**Table 2:** **Odds ratios for the association between a 1-standard deviation increase in leg lean mass and foot pain among men and women in the Framingham foot study, 2002–2008**

	**Model 1** ^**a**^		**Model 2** ^**b**^		**Model 3** ^**c**^	
	**OR**	**95% CI**	**OR**	**95% CI**	**OR**	**95% CI**
All participants	0.85	0.76, 0.96	0.76	0.67, 0.86	0.89	0.73, 1.08
Men	1.03	0.85, 1.25	0.93	0.75, 1.15		
Women	1.08	0.94, 1.24	0.93	0.80, 1.09		

*Abbreviations*: *CI* confidence interval, *OR* odds ratio.

^a^Unadjusted.

^b^Adjusted for age, body mass index.

^c^Adjusted for age, body mass index, sex.

### Foot posture

In the unadjusted model for all participants, a one standard deviation increase in leg lean mass was associated with 23% lower odds of having high arch (OR = 0.77, 95% CI: 0.9, 0.86) and 23% greater odds of having low arch (OR = 1.23, 95% CI: 1.10, 1.38) compared to being in the referent group, but these associations were attenuated after adjustment for age and BMI (Table 3). Following further adjustment for sex, higher leg lean mass was associated with 27% lower odds of having high arch versus being in the referent group (OR = 0.73, 95% CI 0.60, 0.89), but not with having low arch (OR = 1.07, 95% CI 0.88, 1.30). Similarly, in sex-specific models, a one standard deviation increase in leg lean mass was associated with 30% and 19% lower odds of having high arch in men and women, respectively, but not with having low arch (Table 3).

**Table 3:** **Odds ratios for the association between a 1-standard deviation increase in leg lean mass and cavus (High) or Planus (Low) Foot arch (Compared with rectus (Referent) arch), among men and women in the Framingham foot study, 2002–2008**

	**Model 1** ^**a**^				**Model 2** ^**b**^				**Model 3** ^**c**^			
	**Cavus vs. rectus**		**Planus vs. rectus**		**Cavus vs. rectus**		**Planus vs. rectus**		**Cavus vs. rectus**		**Planus vs. rectus**	
	**OR**	**95% CI**	**OR**	**95% CI**	**OR**	**95% CI**	**OR**	**95% CI**	**OR**	**95% CI**	**OR**	**95% CI**
All participants	0.77	0.69, 0.86	1.23	1.10, 1.38	0.93	0.82, 1.06	1.10	0.98, 1.24	0.73	0.60, 0.89	1.07	0.88, 1.30
Men	0.66	0.55, 0.80	1.31	1.10, 1.57	0.80	0.65, 0.98	1.17	0.96, 1.42				
Women	0.70	0.60, 0.81	1.14	0.98, 1.33	0.81	0.69, 0.96	0.95	0.80, 1.13				

*Abbreviations*: *CI* confidence interval, *OR* odds ratio.

^a^Unadjusted.

^b^Adjusted for age, body mass index.

^c^Adjusted for age, body mass index, sex.

### Foot function

For all participants, a one standard deviation increase in leg lean mass was associated with 21% reduced odds of having pronation (OR = 0.79, 95% CI: 0.70, 0.88) and 16% greater odds of having supination (OR = 1.16, 95% CI: 1.04, 1.30), relative to the odds of being in the referent foot function group, in unadjusted analyses (Table 4). Associations were similar after adjustment for age and BMI, but additional adjustment for sex attenuated the results. Among the men, leg lean mass was not associated with foot function. In women, a one standard deviation increase in leg lean mass was associated a modest increased odds of both pronation (OR = 1.11, 95% CI: 0.95, 1.30) and supination (OR = 1.17, 95% CI: 0.98, 1.39), though confidence intervals included the null value.

**Table 4:** **Odds ratios for the association between a 1-standard deviation increase in leg lean mass and pronation or supination (Compared with referent), among men and women in the Framingham foot study, 2002–2008**

	**Model 1** ^**a**^					**Model 2** ^**b**^				**Model 3** ^**c**^			
	**Pronation vs. referent**			**Supination vs. referent**		**Pronation vs. referent**		**Supination vs. referent**		**Pronation vs. referent**		**Supination vs. referent**	
	**OR**	**95% CI**		**OR**	**95% CI**	**OR**	**95% CI**	**OR**	**95% CI**	**OR**	**95% CI**	**OR**	**95% CI**
All participants	0.79	0.70, 0.88		1.16	1.04, 1.30	0.81	0.72, 0.91	1.23	1.09, 1.39	1.07	0.88, 1.29	1.01	0.84, 1.22
Men	0.98	0.81, 1.18		0.90	0.77, 1.06	0.92	0.74, 1.14	0.87	0.72, 1.05				
Women	1.01	0.88, 1.17		1.06	0.90, 1.25	1.11	0.95, 1.30	1.17	0.98, 1.39				

*Abbreviations*: *CI* confidence interval, *OR* odds ratio.

^a^Unadjusted.

^b^Adjusted for age, body mass index.

^c^Adjusted for age, body mass index, sex.

The association of lean mass with foot pain, posture and function were similar after all analyses were repeated using lean mass values adjusted for age-related loss of lean mass between the lean mass and foot assessments (data not shown).

## Discussion

Among our population of older adults, we found that in the overall population higher leg lean mass was associated with lower odds of foot pain, lower odds of pronation, and higher odds of supination, but that these associations were completely explained by sex. Higher leg lean mass was, however, associated with lower odds of high (cavus) arch in the overall cohort after adjusting for sex, as well as in men and women separately. These findings suggest that low muscle mass may be associated with high arch foot posture in the general community of older adults.

We are unaware of any previous reports from popu-lation-based studies of an association between lean mass and foot posture, yet investigations in clinical populations suggest that lower extremity muscle characteristics are determinants of pes cavus. Patients with Charcot-Marie-Tooth disease, an inherited neurological disorder primarily affecting the peripheral nerves in the lower extremities, frequently exhibit pes cavus resulting from weakness in the peroneus brevis and tibialis anterior muscles relative to their natural antagonist tibialis posterior and peroneus longus muscles, respectively [17,18]. Similar imbalances in the peroneal and anterior compartments of the leg muscles have been shown to contribute to cases of pes cavus associated with other pathologies [16]. Additionally, young adults with pes planus demonstrated different sizes and thicknesses of the intrinsic and extrinsic muscles of the foot on ultrasound compared with normal controls [42]. We found that greater overall mus-cle mass in the legs was associated with lower odds of pes cavus. DXA lean mass is, however, unable to assess specific muscles in the leg, thus we are unable to determine if it is an imbalance in antagonist pairs, rather than general decreased muscle mass, which was associated with pes cavus in our study population.

While our results suggest an association between leg lean mass and foot posture, we did not observe an association with foot function. This was not unexpected, as the path of the center of pressure through the foot when walking (measured with the CPEI) represents the summation of several dynamic proximal influences. In particular, variation in the pattern of lumbo-pelvic and thigh muscle activity when walking has been shown to influence foot function and predisposition to foot and ankle injury [43–45], however these variations cannot be evaluated by DXA measure of lean leg mass.

The lack of an association between leg lean mass and foot pain in our study is in agreement with the only other prior study, that we are aware of, to examine muscle mass in relation to foot pain. In a group of 136 adults aged 25–62 years recruited from the community, total skeletal muscle mass was similar between those who did and did not report foot pain, even prior to adjustment for sex [25]. These findings may be difficult to compare to our results as our study population included a relatively large number of older adults. Conversely, in another community-based study of 312 adults aged 60 years and older, participants reporting foot pain had lower ankle dorsiflexion strength than those who did not report pain [6]. The conflict between this prior finding and our current observation may reflect the limitations of lean mass as a measure of muscle status. While lean mass is a highly precise and objective estimate of muscle mass, it is often not an accurate surrogate for muscle strength, particularly among older adults. Although muscle mass is an important determinant of muscle strength [46], the decrease in strength with aging occurs at an accelerated rate compared to the concomitant decrease in mass [41,47]. This uncoupling is due to other age-related changes in muscle, including fat infiltration into muscle tissue [48,49] and denervation of muscle fibers [50]. Direct measures of muscle strength may provide a more accurate assessment of the relation of muscle to foot problems.

Our finding that sex confounded the relations of lean mass with foot pain and function is not wholly unexpected. It is well recognized that women tend to have less lean mass compared to men [51], and prior studies have indicated that women have higher rates of foot pain and extremes in foot posture and function [7,52,53]. Nevertheless, the results from our lone study should not be interpreted as conclusive evidence that lean mass is not associated with foot pain or function.

It is also important to interpret our findings in light of other limitations of our study. First, we were unable to establish a causal relation between lean mass and foot problems because our study was cross-sectional and could not determine whether loss of lean mass preceded foot pain, posture or function, or vice versa. For example, it may be that lower leg lean mass and weakness are a consequence of the smaller range of motion associated with pes cavus [54]. Second, the time difference between our lean mass assessment and foot exam must be acknowledged. Although we repeated all analyses after adjusting lean mass measures for age-related loss of lean mass, we assumed a constant rate of loss over time that was identical across all individuals. It is possible that participants with foot problems may have been less active, which could contribute to an accelerated loss of muscle mass in this group. Thus, we may have underestimated lean mass in those with foot problems, potentially masking any associations in our cohort. Third, since the “legs” region of whole body DXA scans is composed of lean mass of the entire lower extremities, we were unable to separate the lean mass of individual legs to examine leg-specific associations, and we could not specifically measure musculature of the foot. Although foot muscle impairments may be more directly related to foot posture and function, there is evidence to suggest involvement of leg muscles as well [14,17–20]. Fourth, our measures of foot posture and function may have larger measurement error relative to other studies since only one scan was ascertained per participant due to time constraints. Thus, our sex-specific odds ratios may be biased toward null associations and may partially account for the absence of associations of lean mass with foot pain and function. Finally, our study included only Caucasians, limiting the generalizability of our results.

Despite the above limitations, our investigation has important strengths. This was, to our knowledge, the largest population-based study of lean mass and foot pain, posture and function. Our cohort included both men and women, across a wide age range of older adults, from the well-characterized Framingham Study. Additionally, our measures of both foot posture and foot function were objectively ascertained using a pressure mat, as opposed to subjective clinical observation.

## Conclusions

In conclusion, our findings suggest that while muscle mass is not associated with foot pain or function in older adults, reduced muscle mass may contribute to extreme foot posture in older adults, perhaps playing a role in the etiology of physical limitations and disability due to foot disorders. Muscle mass and strength are potentially modifiable and could be considered as targets for intervention to prevent or improve foot problems and consequent physical impairments. Prospective studies that can evaluate the mass and strength of the muscles specific to foot posture and function are needed to gain a better understanding of the etiology of foot problems and the associated consequences.

## Abbreviations

BMI: Body mass index
CI: Confidence interval
CPEI: Center of pressure excursion index
DXA: Dual-energy x-ray absorptiometry
MAI: Modified arch index
OR: Odds ratio
